# Host density and parasitoid presence interact and shape the outcome of a tritrophic interaction on seeds of wild lima bean

**DOI:** 10.1038/s41598-019-55143-5

**Published:** 2019-12-09

**Authors:** Maximilien A. C. Cuny, Juan Traine, Carlos Bustos-Segura, Betty Benrey

**Affiliations:** Institute of Biology, Laboratory of Evolutive Entomology, University of Neuchâtel, Rue Emile-Argand 11, 2000 Neuchâtel, Switzerland

**Keywords:** Ecology, Ecology

## Abstract

The interaction between the seed beetle *Zabrotes subfasciatus* and its parasitoid *Stenocorse bruchivora*, was investigated on seeds of two populations of wild lima bean, *Phaseolus lunatus*. By manipulating the number of beetle larvae per seed and the presence of parasitoids, we determined how factors related to beetle larvae density, the seed in which they feed and the parasitoid, may interact and affect host and parasitoid survival. Results showed that an increase in larval beetle density had a negative impact on beetle performance. This effect cascaded up to parasitoids, high larval density strongly reduced parasitoid emergence. Also, parasitoid presence resulted in faster beetle development and lower female weight. An interactive effect between larval host density and parasitoid presence affected the number of insects that emerged from the seeds. Beetle performance was better in the bean population with the largest seeds, while parasitoid emergence was the lowest in these seeds. This study shows that the impact of parasitoids on seed beetles is contingent on the interaction between density-mediated (direct mortality) and trait-mediated (e.g. non-consumptive) effects. Indirect trait-mediated effects of natural enemies are likely prevalent across insect communities, understanding their role in driving host-parasitoid interactions can have important implications for biological control.

## Introduction

Understanding the effects that parasitoids have on their hosts and their host’s populations, is a long-standing quest in ecological research. Because of their importance in biological control, most studies have focused on how parasitoids reduce host populations through direct mortality and the consequences on the host’s and parasitoid’s population dynamics^1–3^. However, parasitoids can also affect their hosts indirectly through non-consumptive effects^4–7^. Indirect non-consumptive effects are usually non-lethal but can alter the behavior or performance of the host in ways by which their consequences may go beyond the two trophic level interaction and cascade to the whole community^8,9^.

However, the non-consumptive effects of natural enemies are less studied than direct consumptive effects, although they are believed to be very important in shaping ecological interactions^10–14^. For example, Fill *et al*.^15^ tested non-consumptive effects of an aphid parasitoid (*Aphidius colemani*) on non-hosts pea aphids (*Acyrthosiphon pisum*). They found that because of an increase in escape behaviors of pea aphids (i.e. dropping from the plant; reviewed in Humphreys and Ruxton^16^), the parasitoids presence significantly reduced aphid population growth, even without parasitism. In another study, Ingerslew and Finke^17^ showed that parasitoid non-consumptive effects can also impact aphids that do not exhibit dropping behavior but instead, tend to walk away from the parasitizing wasp. Non-consumptive effects of parasitoids on their hosts are more evident for insects that are exposed, such as aphids or caterpillars, where changes in behavior or development can be observed. For concealed insects (i.e. seed beetles), this is less evident and likely one of the reasons why it is rarely documented^18,19^. Seed beetles lay their eggs on the seed coat and upon hatching, larvae burrow inside the seed where they complete their development confined to this closed space^20^. The two most important factors that affect seed beetle performance and survival are the characteristics of the seed in which they develop and the natural enemies that attack them. Physical and chemical characteristics of the host seed are known to greatly affect beetle performance and behavior^21–24^. For example, numerous studies show that when faced with a choice, seed beetles prefer to oviposit on larger than smaller seeds^25,26^. The reason for this is often related to the amount of resource available in larger seeds and the consequences on adult body size and ultimately their fitness^27^. Seed beetles lay several eggs on the same seed, which means that larval competition can be very intense and, as can be expected, is more significant in small seeds^25,26^.

While inside the seed, beetle larvae are frequently attacked by parasitoids^24,28–31^ which often results in the death of the larva (direct effects), but parasitoids can also indirectly impact their host’s performance and survival, through their sole presence, usually by inducing avoidance or defensive behaviors^18,19^. Reciprocally, the performance of parasitoids of seed beetles can also be affected by their host. Host traits responsible for changes in parasitoid performance have received considerable attention in the past decades^1,32–34^. For instance, parasitoids may benefit from a weaker immune system of their host or a slower development, that could prolong the host’s vulnerability^13,24^. In contrast, parasitoid performance can be negatively affected if their host sequesters toxic chemical compounds^24^. Similarly, changes in host size can affect parasitoid’s performance^35,36^. For example, Campan and Benrey^35^ found that the performance of the parasitoid *Stenocorse bruchivora* was better when it parasitized larger beetle larvae in cultivated seeds of *Phaseolus vulgaris* compared to smaller larvae in wild seeds. It is likely that the intraspecific competition among seed beetle larvae is relaxed in these larger cultivated seeds resulting in larger beetles^26^. The interactive effects of host density leading to intraspecific competition and the action of parasitoids have mainly been studied for their effect on host immune responses against endoparasitoids of caterpillars^37–39^. These studies found that an increase in density increases the host’s susceptibility to parasitism, due to a decreased in parasitoid larval encapsulation^37,38^. Yet, for parasitoids of seed beetles, little is known on how host density inside the seed can alter parasitoid responses and performance. For instance, an increase in beetle competition could lead to a negative effect on their larval performance^26^, which could in turn negatively affect parasitoids. Alternatively, higher host density inside the seed could positively benefit parasitoids by facilitating host finding or accessibility. Parasitoids of concealed hosts such as seed beetles, use a combination of chemical, physical and mechanical cues to locate their hosts^1,40–42^. Vibrational cues produced by host feeding and movement may be enhanced as host density increases, facilitating the location of hosts by searching parasitoids^43,44^. However, hosts may sense the presence of searching wasps and reduce their feeding activity (reviewed in Abram *et al*.^7^). These effects of parasitoids on their hosts’ behavior can lead to indirect non-consumptive parasitoid effects on host survival and development that go beyond the mortality effects of direct parasitism.

In the present study, we examined this for the beetle species *Zabrotes subfasciatus* and its larval parasitoid *Stenocorse bruchivora*. We determined (1) the effects of beetle larval density inside the seed on beetle and parasitoid performance and (2) the direct and indirect (consumptive and non-consumptive) effects of parasitoids on beetle performance.

## Materials and Methods

### Study system

*Zabrotes subfasciatus* is a multivoltine seed beetle considered as one of the most important pests of stored beans worldwide^28^. In its native range in southern Mexico and Central America, it attacks wild and cultivated species in the genus *Phaseolus*, among them, *Phaseolus lunatus*, lima bean^45^. Females enter the mature pod and glue their eggs on the seed coat^23^. Few days later, larvae pierce the seed coat and burrow into the seed, where they complete their development until adult emergence^23^. Several eggs can be laid on the same seed, but beetle survival decreases with high larval density^25,26^.

*Stenocorse bruchivora* is one of the main parasitoid species of *Z. subfasciatus*^35^. This solitary ectoparasitoid attacks third and fourth instars and can lay up to 67 eggs throughout their lifespan^28^. As an idiobiont, it paralyzes its host before parasitism, preventing it from feeding and growing, and because its host range is limited to a few species, it is considered a specialist^46^.

### Seed populations and insect rearing

Previous studies with this bean species showed great variation among different populations in chemical and physical seed traits^47,48^. To account for this variation, in our experiments we used seeds from two distinct natural populations located along the Pacific coast in the state of Oaxaca, Mexico (BLO: N16 35.732 W98 46.102, YEL: N16 15.071 W97 48.169), that were previously characterized for their content of cyanogenic glycosides^47^. Populations were 150 km apart from each other. Seeds were collected in 2014 and transported to the lab where they were stored in a cold incubator (13 °C). Because seed size is known to have an effect on beetle oviposition and subsequent survival^28,48^, a random sample of 50 seeds per population was measured with an electronic digital caliper (Vogel, Germany) to the nearest 0.01 mm. Beetles of *Z. subfasciatus* were collected in 2015 (N15 55.330 W97 09.132) and reared in our laboratory on *Phaseolus vulgaris* seeds. *S. bruchivora* parasitoids were obtained from the same location by collecting beetle-infested seeds and reared in our laboratory on *P. vulgaris* seeds infested by 20-day-old *Z. subfasciatus* beetle larvae.

### Experimental set-up

Seeds and insects were kept in the same incubator until the end of the experiment (28/24 °C, 12/12). Seeds from the two populations were randomly distributed among eight plastic jars (8 × 10 cm), four jars for each population, with 100 seeds per jar. We obtained seeds with eggs of *Z. subfasciatus* by placing fifty mated 1-day-old females in each jar. Twenty-four hours later, we checked seeds for eggs with a stereoscopic microscope and selected seeds with one or two eggs, which is common in the wild. Under lab conditions females sometimes lay more than two eggs; we removed excess in egg densities by carefully using fine forceps. This process was repeated for another 24 hours with the seeds that did not receive any eggs until we obtained approximately 300 seeds per plant population with one or two eggs. Few days after egg-laying, we checked that every egg became white and opaque, indicating that eggs had hatched and larvae had entered the seed^49^. Twenty days after beetle oviposition, we placed eight seeds from each population (“BLO” and “YEL”) in a petri dish (9 × 1.5 cm). We used a factorial design with the number of beetle larvae in the seeds (one or two; hereafter, beetle density treatment) and parasitoid presence or absence (hereafter, parasitoid treatment), as treatments (104 petri dishes in total). We set-up the same number of seeds and beetle larvae per petri dish for each treatment (a total of eight seeds and eight beetle larvae per petri dish) while modifying the density of the beetle larvae per seed (one or two per seed). Consequently, in the treatments with one beetle larva per seed, all the eight seeds were infested. On the other hand, in the treatments with two beetle larvae per seed, four seeds were infested by two beetle larvae, and the other four seeds remained uninfested. This design allowed us to offer parasitoids the same number of seeds and hosts yet, varying their distribution.

For the parasitoid treatment, one adult female was introduced in each petri dish when the beetle host larvae were twenty days old. All female parasitoids were naïve, mated and less than five- days old. Wasps were left in the petri dish for three days, after which, each seed from all the treatments was individually transferred to a 1.5 ml Eppendorf tube until insect emergence. We recorded beetle development time (number of days from the first day of egg-counting until the day of emergence), parasitoid development time (number of days estimated from the second day of parasitism until adult emergence), beetle and parasitoid emergence (whether or not a beetle or a parasitoid emerged per each beetle egg, i.e. binomial response). We estimated the number of insects that failed to emerge as adults (both beetles and parasitoids) according to the initial number of beetle eggs. We also measured beetle and parasitoid sex ratio and adult fresh weight (measured in mg with an analytical balance Mettler Toledo XP6, Columbus, Ohio, USA).

### Statistical analyses

All statistical analyses were performed with R (version 3.3.1). Seed size from the two wild lima bean populations was compared using a t-test. In all models, populations were used as covariate and “seed identity” (i.e. each individual seed) nested within petri dish was used as a random factor.

For the analysis of beetle emergence and insect sex ratio we used a generalized linear mixed model (function glmer from the *lme4* package) with a binomial distribution. Explanatory variables were beetle density, parasitoid presence and their interaction. We performed the same model for parasitoid emergence, without parasitoid presence as an explanatory variable. We analyzed the number of insects that failed to emerge from the seeds per petri dish with a GLMM and a Poisson distribution, with parasitoid presence, beetle density and their interaction as explanatory variables. Beetle weight was analyzed using a linear mixed model (function lmer from the lme4 package) following a normal distribution. Beetle density per seed, parasitoid presence and their interaction were used as explanatory variables. The same model was used for parasitoid weight, without parasitoid presence as a variable. Beetle and parasitoid development time were analyzed using cox mixed models (function coxme from the *coxme* package) where we fitted development time according to density per seed, parasitoid presence (only for models concerning beetles) and their interaction. Tukey post-hoc analyses were used for male beetle weight and female beetle development time.

## Results

The analysis on seed size confirmed that seeds from the two wild populations were significantly different in size (Supplementary Fig. S1, DF = 97.1, t = 3.04, *P* = 0.003).

### Effect of beetle density and seed population on beetle performance

**Figure 1:**
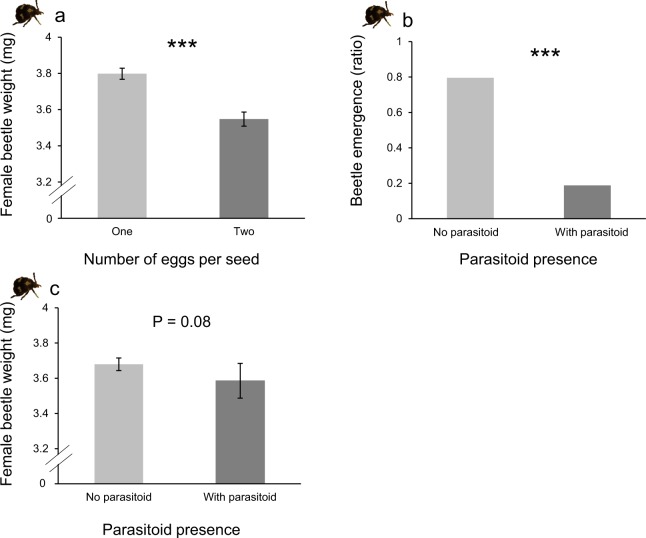
The effect of beetle larvae density and parasitoid presence on beetle performance. (**a**) The effect of beetle larvae density per seed on female weight. The Y axis was cut for clarity purpose. One larva per seed: n = 89, two larvae per seed: n = 95. (**b)** Parasitoid presence on the proportion of adult beetle emergence (total number of emerged beetles divided by the number of beetle eggs on the seed). No parasitoid: n = 416, with parasitoid: n = 416. (**c**) Parasitoid presence on adult female beetle weight. The Y axis was cut for clarity purpose. No parasitoid: n = 151, with parasitoid: n = 33. Asterisks indicate significant differences (***P < 0.001). Bars indicate standard error of the mean.

Adult beetle emergence was not influenced by their larval density in the seeds (DF = 1, Χ^2^ = 0.07, *P* = 0.78). Overall, 22% more beetles emerged from seeds from the BLO population compared to seeds from YEL (Supplementary Fig. S2a, DF = 1, Χ^2^ = 6.8, *P* = 0.009). The development time of both female and male beetles was not affected by the density of beetle larvae in the seed (DF = 1, Χ^2^ = 1.21, p = 0.23; DF = 1, Χ^2^ = 1.19, p = 0.23, respectively) nor by seed population (Females: DF = 1, Χ^2^ = 0.72, p = 0.47; Males: DF = 1, Χ^2^ = 1.56, p = 0.12). The interaction between seed population and beetle density had a significant effect on male beetle weight (DF = 1, F = 4.36, p = 0.037), but not on females: males emerging from BLO seeds with two beetles were significantly heavier than males emerging from YEL seeds (DF = 3, F = 16.17, *P* = 0.001). Females emerging from seeds with only one egg and from seeds of the population BLO were significantly heavier than females from seeds with two eggs (7% heavier, Fig. 1a, DF = 1, F = 14.38, *P* < 0.001) and from seeds of the population YEL (5% heavier, Supplementary Fig. S2b, DF = 1, F = 4.74, *P* = 0.029). Beetle sex ratio was not affected by any of the treatments (beetle density per seed: DF = 1, Χ^2^ = 0.159, *P* = 0.69; seed population: DF = 1, Χ^2^ = 0.023, *P* = 0.88).

### Effect of parasitoid presence on beetle performance

**Figure 2:**
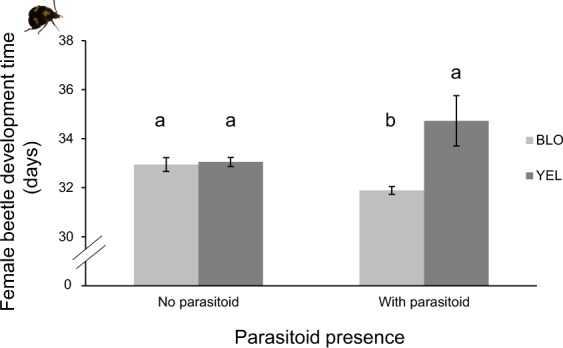
The effect of the presence of parasitoids on the development time of female beetles in two seed populations of lima bean. For beetles developing in seeds from BLO population: no parasitoid: n = 71, with parasitoid: n = 18. In seeds from YEL population: no parasitoid: n = 80, with parasitoid: n = 11. Error bars represent the standard error of the mean. Significant differences are indicated by different letters (*P* = 0.003). The Y axis was cut for clarity purpose.

**Figure 3:**
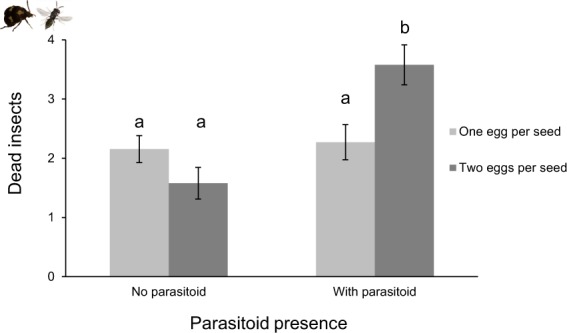
Effect of parasitoid presence on the mean number of insects (beetles and parasitoids) that did not emerge from seeds of two lima bean populations. n = 26 for each treatment. Bars represent standard error of the mean. Significant differences are indicated by different letters (*P* = 0.002).

As it was expected, beetle emergence was 76% lower when parasitoids were present (Fig. 1b, DF = 1, Χ^2^ = 115.9, *P* < 0.001). Parasitoid presence had a marginal negative effect on the weight of adult female beetles (Fig. 1c, DF = 1, F = 3.12, *P* = 0.08) but did not influence the weight of adult male beetles (DF = 1, F = 0.016, *P* = 0.9). We found a significant interaction between parasitoid presence and seed population (Fig. 2, DF = 1, Χ^2^ = 3.27, p = 0.001) on female beetle development time: parasitoid presence significantly reduced the development time of female beetles, although this was only the case in BLO population (Fig. 2, DF = 3, Χ^2^ = 15.67, p = 0.003). The reverse pattern was observed in the other population, but it was not statistically significant (p = 0.4). However, parasitoid presence did not affect male beetle development time (DF = 1, Χ^2^ = 0.75, p = 0.45). The number of dead insects, measured as the number of insects that failed to emerge from the seeds (comprising non-parasitized and parasitized beetles, as we cannot discern from these two categories) was affected by the interaction between parasitoid presence and beetle density (DF = 1, Χ^2^ = 8.626, *P* = 0.003). When parasitoids were present, the number of dead insects (expected insects per petri dish, both beetles and parasitoids, that failed to emerge as adults) was higher in seeds with two eggs than in seeds with one egg (Fig. 3, DF = 1, Χ^2^ = 9.96, *P* = 0.002). Parasitoid presence had no significant effect on the sex ratio of emerged adult beetles (DF = 1, Χ^2^ = 2.716, *P* = 0.099).

### Effect of beetle density and seed population on parasitoid performance

**Figure 4:**
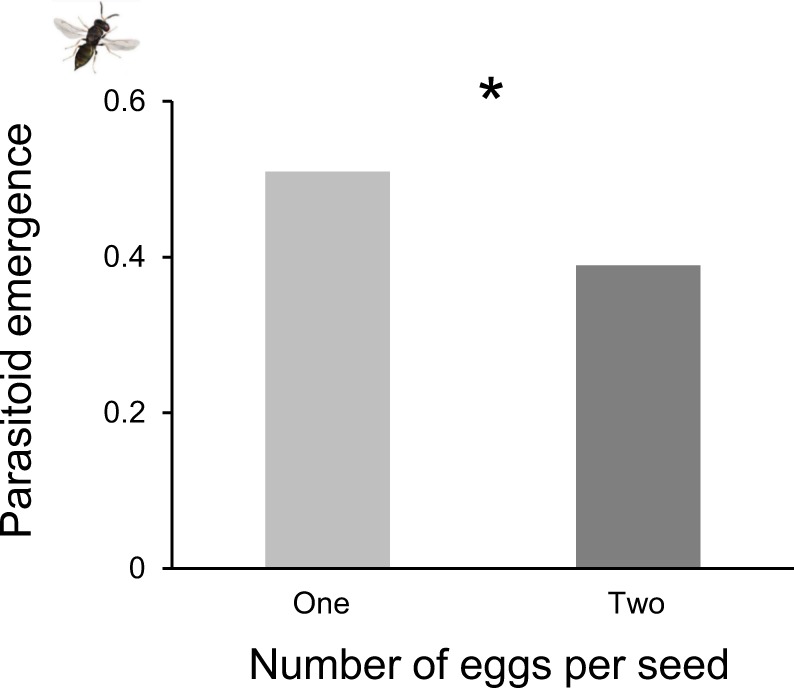
The effect of beetle larvae density per seed on parasitoid emergence (total number of parasitoids emerged divided by the total number of available hosts). One beetle larva per seed: n = 208, two beetle larvae per seed: n = 208. Asterisks indicate significant results (*P < 0.05).

Parasitoid emergence was 30.8% higher when parasitoids were offered seeds with only one beetle larva compared to seeds with two larvae (Fig. 4, DF = 1, Χ^2^ = 5.66, *P* = 0.01) and was 36% higher when they developed in seeds from YEL compared to BLO (Supplementary Fig. S2c, DF = 1, Χ^2^ = 7.41, p = 0.006).

The development time of female and male parasitoids was not affected by the seed population (DF = 1, Χ^2^ = 1.42, *P* = 0.15; DF = 1, Χ^2^ = 0.63, *P* = 0.53; respectively), nor by the density of beetle larvae in the seed (DF = 1, Χ^2^ = 0.65, *P* = 0.51; DF = 1, Χ^2^ = 0.27, *P* = 0.79; respectively). Beetle larval density per seed had no significant effect on parasitoid weight, for both males (DF = 1, F = 0.005, *P* = 0.94) and females (DF = 1, F = 1.98, *P* = 0.16). Similarly, parasitoid weight was not affected by the population of the seed in which they developed (males: DF = 1, F = 0.06, *P* = 0.8; females: DF = 1, F = 0.51, *P* = 0.47). Parasitoid sex ratio was not affected by beetle density per seed (DF = 1, Χ^2^ = 0.336, *P* = 0.56) nor by seed population (DF = 1, Χ^2^ = 0.01, *P* = 0.98).

## Discussion

In this study we investigated the interactive effects of beetle larval density inside the seed and the presence of parasitoids on a tritrophic interaction with two wild lima bean populations. We found that beetles performed better when only one larva developed inside a seed, and these effects cascaded up to the third trophic level strongly reducing parasitoid emergence. Our results also reveal both, direct and indirect effects of parasitoids on beetle performance. When beetle-infested seeds were exposed to parasitoids, fewer beetles emerged (due to direct mortality) and female beetles developed faster in one of the seed populations and were marginally of lower weight (indirect effects) than those that developed in seeds not exposed to parasitoids. Moreover, we found an interactive effect between density per seed and parasitoid presence on the total number of insects that successfully emerged from the seeds. Finally, beetle and parasitoid performance was different in seeds from the two bean populations.

When one larva developed alone inside the seed, emerging females were on average 7% heavier than females from seeds with two beetle larvae. Because males are smaller than females^23^, they likely need less resources to develop and are not as much affected by larval competition. This is supported by results from a previous study in which the negative impact of intraspecific larval competition inside the seed was higher when female beetles developed in smaller wild seeds compared to larger cultivated seeds, whereas males were not affected^26^. Differences in female weight or size can have major consequences on their fitness, as it is known that fecundity is higher in larger than in smaller females^23,28^. We however, did not find any significant effect of beetle larval density on development time and adult emergence. This result differs from those of a number of studies that have reported that beetle competition can have negative effects on their development time and survival^25,26,50^. These contrasting results may be due to the higher larval densities used in previous studies compared to those in our study, in which we simulated egg densities commonly found in nature on these small wild seeds (one or two eggs). The difference in seed size between these two populations was relatively small (on average, 6.7 mm and 6.5 mm), yet we still found that beetle performance (weight and emergence) was higher in larger seeds of the BLO population than in smaller seeds of YEL. Whether these small differences in seed size are biological relevant, should be investigated. Other studies have also reported differences in performance of this beetle species on seeds from different natural populations that vary in size^24,26^. On one hand, larger seeds offer more resource for developing beetles^26^. On the other hand, in the case of seeds of *P. lunatus*, larger seeds have a higher protein content than smaller seeds, which results in better beetle performance^36^.

As for the parasitoids, when more than one beetle larva developed in the seed, parasitoid weight and development time were not affected, but parasitoid emergence was strongly reduced (about 25% lower). Additionally, we found an interactive effect of beetle density inside the seed and parasitoid presence on the proportion of adult insects that successfully completed development inside the seed (both beetles and parasitoids): in the presence of parasitoids, fewer insects emerged from seeds that carried two beetle larvae. Therefore, it is likely that parasitized larvae are more prone to mortality when beetle competition increases^51^. The effect of host density on parasitism rates has been studied extensively in the context of parasitoid functional responses^52–56^. These studies have mainly examined the number of hosts parasitized as a function of varying host densities. In our study, the total number of hosts available was kept constant, but their distribution per seed was different (one or two beetles per seed), which resulted in a negative impact on beetle performance, most likely due to larval competition. Competition (intra and interspecific) is regarded as one of the main factors shaping ecological communities^57,58^. Yet, its role in driving host-parasitoid interactions is poorly studied^37–39^. Kraaijeveld and Godfray^37^ reared different lines of *Drosophila melanogaster* with and without parasitoids, in order to select host lines with a better ability to encapsulate parasitoid eggs. Then, they exposed the lines to different levels of intraspecific competition, and showed that *D. melanogaster* lines that were selected for an increase in parasitoid egg encapsulation had a lower survival in conditions of high competition, compared to lines that were not selected for egg encapsulation. Their study revealed a trade-off between host traits involved in host competition and traits related to defense against parasitoids, indicating that these traits may be more closely related than previously considered.

Contrary to the findings on beetle emergence, a higher proportion of parasitoids emerged from seeds of YEL than from seeds from the BLO population. This is somehow unexpected as several studies have shown that parasitoid performance is positively correlated with host performance^59^, and here we found that emergence and female beetle weight were higher on BLO population. For endoparasitoids a larger and better quality host could better encapsulate the parasitoid eggs^1,60,61^. However, for ectoparasitoid species like *S. bruchivora* it is not known the extent to which size (and quality) of their host can influence their immune response and their ability to overcome parasitism. Another seed trait that could differentially affect the beetles and their parasitoids, is secondary compounds. Wild lima bean seeds have high levels of cyanogenic glycosides (CNGs) that are known to be well tolerated by *Z. subfasciatus*^47^, but perhaps not the case for their parasitoids. Although this has not been investigated in this particular system, several studies have shown that secondary compounds that are not harmful for the herbivore can negatively affect their parasitoids^59,62,63^. Lastly, the smaller size of seeds from the YEL population, could have facilitated parasitoid oviposition, indicating a direct interaction between seed traits and parasitoids. For example, for gall insects it has been shown that parasitoids are more successful attacking smaller galls because they are easier to reach^64^. This idea however, has not been investigated for parasitoids of seed beetles.

Overall, parasitoids had a two-pronged effect on the beetle host. As could be expected, they drastically reduced the emergence of beetles via direct parasitism and hence, mortality. But interestingly, parasitoid presence had an indirect effect on the development time of non-parasitized female beetles, and this effect was different in the two bean populations. In the BLO population, female beetle developed 3% faster (pupated 24hrs earlier) in the presence of parasitoids than female beetles in the YEL population. In a recent study, Stephan *et al*.^14^ showed that plant genotype can influence the strength of omnivorous predatory bug non-consumptive effects on their preys. They found that omnivorous predators spent more time on higher quality plants, which in turn increased their non-consumptive effects on herbivorous beetles. We also found a marginal negative (p = 0.08) effect of the presence of parasitoids on female beetle adult weight. In accordance with our results, Zaugg *et al*.^13^ studied the interaction between a related beetle species, *Acanthoscelides obtectus* and the parasitoid *Dinarmus basalis*. In the presence of parasitoids, emerged (non-parasitized) female beetles developed faster and weighed less than emerged females that had no contact with parasitoids. They hypothesized that beetle larvae can sense vibrational signals produced by the foraging parasitoids. In response, they reduce their feeding activity in order to limit their own vibrations and pupate earlier. This type of host defensive behavior has been shown in several species of leafminers^43,65,66^. However, very little is known about vibrations perceived by beetle larvae inside the seed. Non-consumptive effects of predators and parasitoids have been shown to have important consequences on herbivore population dynamics^5,7,11,67–69^. For instance, Thaler and Griffin^70^ showed that the negative impact of a predatory stinkbug (*Podisus maculiventris*) on the feeding activity of an herbivorous insect prey (*Manduca sexta*) was approximately the same when they removed the predator’s rostra, preventing them from killing their prey. It should be noted that although we found evidence for faster development and lower weight in female beetles that emerged from seeds exposed to parasitoids, we cannot exclude the possibility that this is not the result of non-consumptive effects. An alternative hypothesis would be that parasitoids differentially choose larger beetle larvae, so that the one beetle that still emerged from the seeds was the smaller one from the beginning. With our present data, we cannot disentangle non-consumptive effects from parasitoid differential host selection. However, two lines of evidence indirectly refute the alternative hypothesis of differential host selection. First, if parasitoids preferentially parasitize larger hosts, as females are larger than males, more females should have been parasitized resulting in a beetle male biased sex ratio. This was not the case, the sex ratio of emerging beetles was not significantly different from 50: 50. Secondly, in the treatment seed with one larva with parasitoid, not all larvae were parasitized and adult beetles emerged from these seeds. The weight of these beetles was lower that the weight of beetles that emerged from seeds with one larva and no parasitoid. To us this indicates that the mere presence of parasitoids resulted in smaller beetles.

In conclusion, our findings indicate that the outcome of the tritrophic interaction among bean seeds, beetles and their parasitoids may stem from the combined effect of density-mediated (larval beetle density) and trait-mediated effects (beetle performance influenced by parasitoid activity, and possibly non-consumptive effects) on beetles and their parasitoids. The lower parasitoid emergence in seeds with two beetle larvae compared to one, suggests that if considered as a biological control agent, *Stenocorse bruchivora* should preferentially be used for large seed species in which the negative effect of beetle competition is reduced^26^. Non-consumptive predator effects are likely prevalent across insect communities. Yet, their consequences on shaping community structure and driving host-parasitoid interactions are not well understood. Our results highlight the need to jointly consider density and trait-mediated effects in host-parasitoid studies. In agricultural systems, a better understanding of the traits that enhance the effects of parasitoids on their hosts, could improve their use as biological control agents. Future work should investigate the specific mechanisms responsible for these effects and how they vary across bean populations and cultivated varieties.

## Supplementary information

Supplementary information 1

Supplementary information 2
