# Supplementary material for: Host density and parasitoid presence interact and shape the outcome of a tritrophic interaction on seeds of wild lima bean
Source: Sci Rep. 2019 Dec 9;9:18591. doi: 10.1038/s41598-019-55143-5 (PMC6901471; doi:10.1038/s41598-019-55143-5)
Supplement: Supplementary file 1 — Supplementary information 1 [file 41598_2019_55143_MOESM1_ESM.pdf]

Host density and parasitoid presence interact and shape the outcome of a tritrophic interaction on seeds of wild lima bean

Maximilien A.C. Cuny, Juan Traine, Carlos Bustos-Segura & Betty Benrey

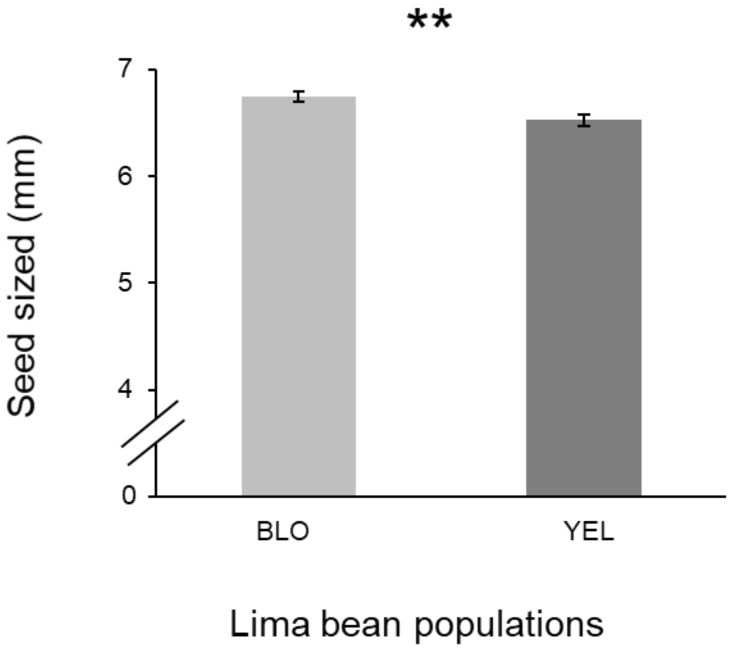

Supplementary figure S1. Mean size of seeds from two populations. n = 50 for each population. Asterisks indicate significant results (**: $P < 0.01$). Bars indicate standard error of the mean.
