# Supplementary material for: Host density and parasitoid presence interact and shape the outcome of a tritrophic interaction on seeds of wild lima bean
Source: Sci Rep. 2019 Dec 9;9:18591. doi: 10.1038/s41598-019-55143-5 (PMC6901471; doi:10.1038/s41598-019-55143-5)
Supplement: Supplementary file 2 — Supplementary information 2 [file 41598_2019_55143_MOESM2_ESM.pdf]

Host density and parasitoid presence interact and shape the outcome of a tritrophic interaction on seeds of wild lima bean

Maximilien A.C. Cuny, Juan Traine, Carlos Bustos-Segura & Betty Benrey

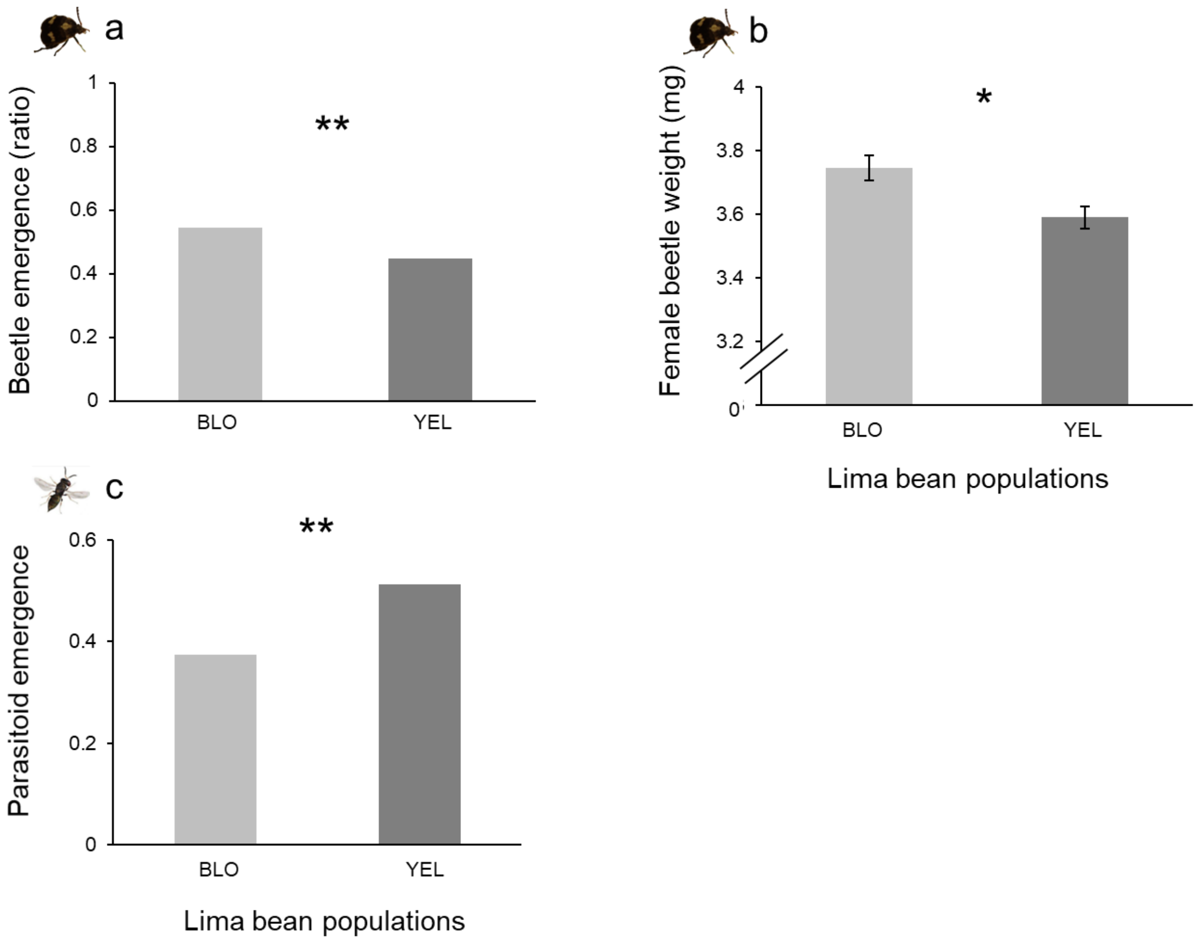

Supplementary figure S2. Effect of lima bean population on beetles and parasitoids. **a** The effect of seed population on the proportion of beetle emergence (total number of emerged beetles divided by the number of eggs). BLO population: n = 384, YEL population : n = 448. **b** The effect of seed population on female beetle weight. The Y axis was cut for clarity purpose. BLO and YEL populations: n = 92. Bars are standard error of the mean. **c** The effect of seed population on parasitoid emergence (total number of parasitoid emerged divided by the total number of available hosts). Lima bean populations: BLO: n = 192, YEL: n = 224. Asterisks indicate significant results (*: $P < 0.05$, **: $P < 0.01$).
